# Embryotoxic effects of diflubenzuron active ingredient and commercial formulations: comparative insights from zebrafish model

**DOI:** 10.1007/s10646-026-03078-9

**Published:** 2026-04-06

**Authors:** Amanda de Azevedo Valle, Rafaella Silva Brito, João Pedro Camargo Mitne, Victoria Marcella Ferreira Almeida, Aline Isabella de Souza, Flavio Kiyoshi Tominaga, Aline Garnevi Fávero, Maria Célia Sanches Oliveira Luz, Anna Virginia Pike, Maysa Zungolo Burrattino, Fábio Kummrow, Bruno Fiorelini Pereira

**Affiliations:** 1 Experimental Danio rerio Facility (BEDAR), Universidade Federal de São Paulo - campus Diadema, Diadema, Brazil; 2 Departament of Pharmaceutical Sciences, Universidade Federal de São Paulo - campus Diadema, Diadema, São Paulo, Brazil; 3 Departament of Biological Sciences, Universidade Federal de São Paulo - campus Diadema, Diadema, São Paulo, Brazil

**Keywords:** Benzoylphenylurea, Zebrafish embryos, Larvicide, Morphology, Teratogenicity, Pigmentation

## Abstract

Diflubenzuron (DFB) is a larvicide widely incorporated into pesticide product formulations (PPFs). In addition to the active ingredient (AI), PPFs contain inert substances and adjuvants that may interfere with the AI toxicity. This study aims to compare the embryotoxicity of a veterinary powder (VF; 3% DFB) and an agricultural water-soluble pellet (AF; 80% DFB) towards DFB, referred to as AI (purity > 99%). Zebrafish (*Danio rerio*) embryos were exposed to AI and both formulations at low (0.025 and 0.125 mg L^− 1^ of AI); intermediate (0.25 and 1.25 mg L^− 1^ of AI); and high (2.5 and 10 mg L^− 1^ of AI) concentrations, as well as a reconstituted water (fish medium) and DMSO (0.4% v/v) controls. Survival, hatching rates, pigmentation, and morphological biomarkers were monitored over 120 h post-fertilization (hpf). No significant effects on survival or hatching rates were observed. Morphological abnormalities, such as notochord deformities and yolk sac opacity, occurred at all concentrations, differing in frequency and intensity. AI showed stronger effects at lower and intermediate concentrations, whereas AF and VF exhibited stronger effects at higher concentrations. Oxygen consumption did not differ significantly from controls. Regarding morphometry, AF induced more pronounced alterations than both PPFs, mainly at intermediate and high concentrations, affecting sensory, physiological, skeletal, and muscle parameters. VF caused minor changes, AI showed no significant effects, and AF presented greater toxicity. AF caused an increase while AI and VF resulted in a slight decrease in body pigmentation. Our findings suggest that PPFs may enhance toxicity by increasing the AI solubility.

## Introduction

Insecticides are any toxic substances used to eliminate or control insect populations, including ovicides and larvicides (Araújo et al. 2023). The excessive use of synthetic pesticides, including insecticides, has contaminated unintended areas over the years, harming non-target organisms and disrupting ecosystems (Albaseer et al. 2025). Thus, since the 1990s, the search for active ingredients (AI) with more specific modes of action and less hazardous to both the environment and biota has intensified. In this context, unconventional insecticides emerge that can interfere with the growth, development, reproduction, and metamorphosis processes of insects. This group of insecticides is known as insect growth regulators (IGRs) (Rezende-Teixeira et al. 2022). The IGRs may or may not induce toxic effects, but they cause abnormalities in insect development, impacting their survival. In general, they have low toxicity to mammals and are effective in controlling several species of arthropods (Rezende-Teixeira et al. 2022; Sankar and Kumar 2023).

Among several available IGRs, diflubenzuron (DFB) was the first chitin synthesis inhibitor (CSI) marketed as an insecticide, introduced in 1975 under the trade name Dimilin^®^ (Sankar and Kumar 2023; Kummrow and Pereira 2024). The DFB is a larvicide of the benzoylurea class widely used in public health programs for vector mosquito control, in veterinary medicine, and in agriculture due to its selectivity and for presenting lower risks of damage to non-target species compared to other classes of synthetic insecticides such as pyrethroids, organophosphates, and carbamates (Sousa et al. 2020; Pener 2021; Sankar and Kumar 2023; Kummrow and Pereira 2024).

The larvicide DFB shows strong persistence in marine sediments near aquaculture sites, where measured concentrations consistently exceed the Norwegian environmental quality standard. It can remain detectable for many months after treatment, reflecting slow degradation and strong binding to organic-rich sediments (Parsons et al. 2021). Samuelsen et al. (2015) found that DFB applied to sediments remained largely unchanged over 24 weeks, demonstrating high stability with no chemical, microbial, or leaching degradation. The Environmental Protection Agency (USEPA) data indicate that DFB is hydrolytically and photochemically stable, degrading slowly under high pH or natural light. It persists moderately under anaerobic conditions, producing degradation products that accumulate in sediments and water, while dissipation is faster in aerobic water–sediment systems (USEPA 2009). However, information on the persistence of DFB in fish and across other aquatic compartments is still limited.

The DFB-based pesticide product formulations (PPFs) are widely used to control arthropods in livestock, aquaculture, agricultural pest management, and disease-vector mosquitoes (Samuelsen et al. 2015; Hannisdal et al. 2020). A PPF rarely contains only the AI. PPFs are usually mixtures of one or more AI and other substances referred to as inert, adjuvants, or co-formulants (Nagy et al. 2020). These components are added to the AI to enhance its chemical and physical efficacy, thereby improving dissolution, stability, absorption, and pesticidal action (Pereira et al. 2009; Nagy et al. 2020). Nevertheless, in the context of commercial formulations, studies documenting its environmental occurrence and actual commercial use remain limited. In general, after application of PPFs, the AI is released from the formulation; however, the release rate depends on factors such as the type of formulation and environmental conditions (Kovačević et al. 2022). However, it is important to note that formulation components considered inert may contribute to the PPF’s overall toxicity by promoting the entry of the AI into organisms, and in some cases, due to their own biological activity, they may be as toxic or even more toxic than the AI (Nagy et al. 2020; Stejskal et al. 2021).

The zebrafish is a well-established model in toxicological research, frequently used to study the effects of pollutants on biological systems. This model has scientific validation and regulatory recognition for assessing adverse effects across different levels of biological organization (Scholz et al. 2008; OECD 2013; MacRae and Peterson 2015). This organism model has 70% of its genes orthologous to humans; thus, it is an ideal model for investigating chemical impacts on vertebrates (OECD 2013; Braga et al. 2024). Zebrafish exhibits physiological and developmental characteristics similar to those of more complex vertebrates, including mammals (Kelkar et al. 2014; Braga et al. 2024). Furthermore, their high fecundity, external fertilization, transparent embryos, and rapid embryonic development allow for efficient high-throughput testing, making them suitable for generating reliable and timely results (Howe et al. 2013; OECD 2013; Aleström et al. 2020).

Although DFB primarily targets chitin synthesis in arthropods, increasing evidence indicates that fish, especially during early development, can be sensitive to this compound. Studies using zebrafish have shown that DFB exposure induces developmental defects in embryos, including increased oxidative stress and apoptosis at concentrations of 0.5–4.5 mg L^− 1^ (Han et al. 2022). Another study found that exposures above 1.25 mg L^− 1^ reduced survival and delayed hatching in zebrafish embryos, and mixtures with other pesticides increased malformations (Teixeira et al. 2024). Chronic exposure from early developmental stages also altered behavior and oxidative stress-related gene expression in juvenile zebrafish (Teixeira et al. 2025).

Although DFB occurs frequently in various environments, previous studies have examined the toxicity of AI (Fischer and Hall Jr 1992; Han et al. 2022), but the effects of its DFB-based PPFs are not yet fully understood. These PPFs may lead to complex interactions that modify the DFB toxicity, highlighting the need for further investigation. Thus, we hypothesize that commercial formulations modulate toxicity differently from the active ingredient. In this study, we aimed to compare the toxicological effects of the AI and two DFB-based PPFs on zebrafish embryos and larvae, providing insights into the potential impacts on the developmental and respiratory effects of these commonly used PPFs.

## Materials and methods

### Chemicals

Diflubenzuron (C_14_H_9_ClF_2_N_2_O_2_; MW = 310.7 g mol^− 1^; *N*-[(4-chlorophenyl)carbamoyl]-2,6-difluorobenzamide; CAS Number 35367-38-5) was obtained from (Sigma-Aldrich; purity > 99%), and hereafter referred to as AI, since it corresponds to the active ingredient evaluated in this study. Two DFB-based PPFs were selected for this study. The first, intended for veterinary use (referred to as VF), is available as a powder and is administered to cattle along with salt, containing 3% of the IA. The second, designed for agricultural use (referred to as AF), is sold as water-soluble pellets for dispersion and spraying, containing 80% of the IA.

## Zebrafish maintenance and reproduction

Adult zebrafish from the Tübingen (TU) strain were maintained and bred at the animal facility of the Federal University of São Paulo (Unifesp) – São Paulo *campus*. All procedures involving the maintenance or use of animals for scientific research were conducted in compliance with National Council for the Control of Animal Experimentation Normative Resolution No. 61/2023 (CONCEA 2023). All the experimental protocols were previously approved by the Unifesp Ethics Committee for Animal Use (CEUA nº 5220291122). The animal’s health was monitored daily, and the adults were kept in 3 L polycarbonate tanks (Alesco^®^) with a recirculating water system (Alesco^®^) under controlled conditions: pH 7.0 ± 0.5, conductivity of 6 µOsm, toxic ammonia (0 ppm), nitrite (0 ppm), temperature (28 ± 1 °C) and photoperiod (14/10 light: dark) (OECD 1992). The animals were fed three times daily with commercial flake food (Alcon^®^ Colours, 42% of protein) and *Artemia salina* nauplii (*ad libitum*) (twice a day) (*Artemia salina* do RN^®^).

For reproduction, adults (6–12 months old) were placed in breeding tanks with a 1:1 male-to-female ratio. The fish were physically separated for 12 h, allowing only visual and chemical interactions. Afterward, the partition was removed, and the fish were mated for one h. After mating, eggs were collected from the tanks, pooled into Petri dishes, and examined under a stereomicroscope (ZEISS Stemi 305). It was evaluated for blastula formation at 3 h post-fertilization (hpf) and verified fertilization.

### Fish embryotoxicity test (FET)

The acute toxicity test was carried out using an extended FET design (120 h) based on the OECD Guideline No. 236 (Fish Embryo Acute Toxicity; OECD 2013), with modifications. After breeding, the embryos were placed in a Petri dish with prepared water (Sea Salt Ocean Tech^®^ prepared in reverse osmosis water at pH 7.0, pH was corrected using sodium bicarbonate) and selected for viability (4–28 cell stage) using a stereomicroscope, following the criteria established by Lammer et al. (2009). Viable embryos at three hpf (*n* = 20 animals per treatment/replicate) were exposed to six nominal concentrations (0.025, 0.125, 0.25, 1.25, 2.5, and 10 mg L^− 1^ of the AI), as well as to a water control (WC, prepared water), and a solvent control (SC, DMSO at 0.4%). The concentrations of VF and AF were prepared to maintain the respective concentration of AI. DFB is a substance practically insoluble in water (0.08 mg L^− 1^), and it exhibits high solubility in solvents such as DMSO (10 g L^− 1^) (Sun et al. 2015). Its formulations, however, were designed to be administered orally in solid form (VF) or as a spray in aqueous medium (AF). Our preliminary assays, conducted before the toxicity tests, showed that both formulations were insoluble in water; moreover, VF displayed insoluble particles in both water and DMSO. To standardize exposure conditions and enhance DFB solubility, the AI and its PPFs were prepared in DMSO. Given the insoluble nature of DFB in water, the maximum solvent concentration established for the experiment was 0.4%, which is above the concentration considered ideal for biomarkers sensitive to DMSO (0.01%) (OECD 2019), but still regarded as acceptable for morphological biomarkers (Hoyberghs et al. 2021). The DFB solubility and the DMSO concentration represent a limitation for this study. Therefore, we compared the tested concentrations with both the water control and the DMSO control. The nominal concentrations described were chosen based on the dosage recommended by the WHO for use as a larvicide, which is 0.25 mg L^− 1^ (WHO 2022). Concentrations above and below were tested while respecting the solubility limit.

The exposure was conducted in 24-well microplates, with one embryo per well. The plates were maintained in a biochemical oxygen demand (BOD) incubator under static conditions (no medium renewal), with controlled temperature (28 ± 1 °C) and photoperiod (14/10 light: dark). A modification of the temperature suggested by OECD No. 236 (26 ± 1 °C) was used in this study to ensure complete development of zebrafish embryos so that most endpoints could be analyzed within 120 hpf (Miller et al. 2025). The experiments were performed in triplicate. Toxicity endpoints were observed daily (every 24 h) during 120 h using a stereoscopic binocular microscope (ZEISS Stemi 305). Survival rate, hatching rate, and frequency of morphological abnormalities (circulatory changes, integumentary alterations, skeletal disorders, or yolk sac abnormalities) were daily analyzed for all treatments (OECD 2013; Pereira et al. 2019). Morphological abnormalities observed during exposure were classified according to Pereira et al. (2019) and Von Hellfeld et al. (2020). Heart rate was measured in 96 hpf, however data were excluded because solvent interference prevented reliable measurement.

### Image acquisition

Embryos at 120 hpf were euthanized with tricaine (800 mg L^− 1^) and fixed in 10% formaldehyde for 24 h. The samples were then immersed in 70% ethanol and stored at 4 °C until the time of analysis. Each sample was photographed using a binocular stereomicroscope (ZEISS Stemi 305, 2.5 x magnification). The larvae were transferred to slides with conductive gel (Mercur^®^) to facilitate the procedure. Twenty larvae were photographed for each concentration, one lateral and one dorsal photo per larvae, for a total of 40 photos per concentration for each formulation.

### Body pigmentation analyses

The images were acquired, and subsequently, the pigmentation was analyzed based on previous studies (Hinz et al. 2012; Grisola and Fuentes 2017). To analyze pigmentation in each specimen, ImageJ software (version 1.54k) was used to quantify and assess the overall distribution of pigmentation. Pigmentation of the larvae was analyzed using ImageJ. Briefly, the Color Deconvolution plugin was applied to separate the pigment signal, followed by thresholding to identify the dark-stained areas. The images were then binarized, and the total area of the pigmented regions was measured and summed to quantify overall pigmentation.

### Morphometric analysis

**Fig. 1:**
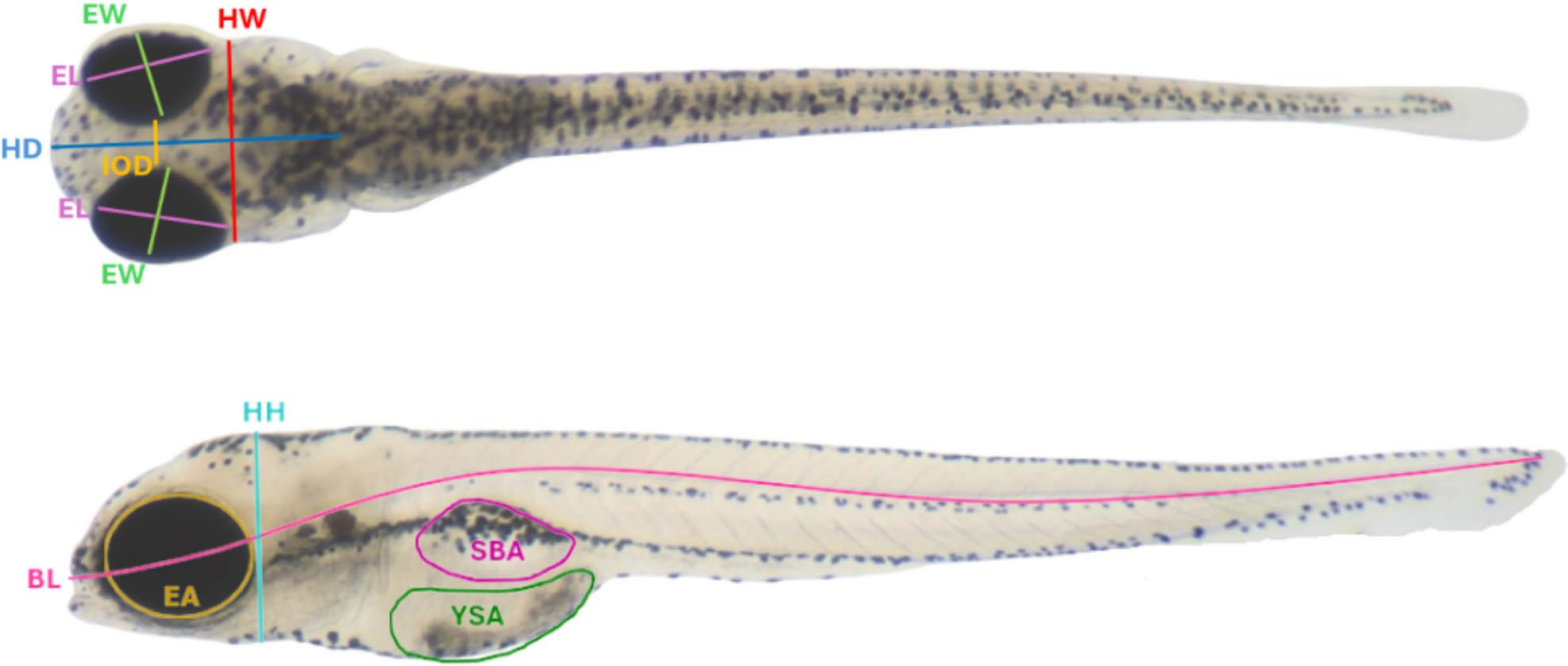
Morphometric parameters were measured in each 120 hpf zebrafish larvae, including sensory (EA, IOD, EL1, EL2, EW1, and EW2), physiological (YSA and SBA), skeletal (BL), and muscular (HH, HW, and HD) traits. EA = Eye area; IOD = Interocular distance; EL1 = Left eye length; EL2 = Right eye length; EW1 = Left eye width; EW2 = Right eye length; YSA = Yolk sac area; SBA = Swim bladder area; BL = Body length; HH = Head height; HW = Head width; HD = Head depth

Following image acquisition, zebrafish larvae at 120 hpf were subjected to morphometric analysis using ImageJ software (version 1.54 K). The Oval and Freehand Selection tools were used to measure areas, while the Straight tool was used to measure length and height. Morphometric parameters were categorized into four groups (sensory, physiological, skeletal structural, and muscular structural), and included the following measurements: eye area (EA), interocular distance (IOD), left eye length (EL1), right eye length (EL2), left eye width (EW1), right eye width (EW2), body length (BL), head height (HH), head width (HW), head depth (HD), yolk sac area (YSA), and swim bladder area (SBA) (Fig. 1). The measurements were adapted from Martínez et al. (2019) and Ribeiro et al. (2020).

### Oxygen consumption

Basal oxygen consumption was measured using an Oxygraph+ system (Hansatech Instruments^®^). Based on the obtained results, zebrafish embryos up to 4 hpf were exposed to the concentrations that demonstrate the most significant effects on the other endpoints evaluated (0.025, 0.125, and 0.25 mg L^− 1^ of the AI), as well as to a water control and a solvent control until 168 hpf (*n* = 30 embryos per treatment). For this analysis, exposure was extended to 168 hpf (Gore and Burggren 2012) so that O_2_ consumption would be high enough to be accurately detected in a pool of 10 larvae, and it would not be necessary to increase the number of embryos exposed, thus following the ethical principles of animal use (3Rs). At the end of the exposure period, a pool of 10 larvae from each replicate was used to quantify oxygen consumption, using a new medium containing only prepared water (fish medium). Larvae with morphological alterations were not included in this assay. For each pool, O_2_ consumption was measured over 5 min, and the rate of change (nM mL^− 1^) was calculated within a representative section of the consumption curve between 3 and 4 min at 30 s intervals.

### Statistical analysis

Statistical analyses were performed using Jamovi (version 2.3.7) and GraphPad Prism (version 8). The Levene test was applied to assess variance homogeneity (homoscedasticity), and the Shapiro-Wilk test was used to evaluate the normality of the data. Mortality and hatching data were organized into Kaplan-Meier curves and compared using the Log-Rank test. Other quantitative results were analyzed using ANOVA (with Tukey post-hoc test) for parametric data, or Kruskal-Wallis and Dwass-Steel-Critchlow-Fligner (DSCF) tests for non-parametric data. Sample sizes were based on OECD No. 236 (OECD 2013) and a previous study (Ribeiro et al. 2020). Level of significance was defined as α < 0.05. To compare the AI and its PPFs, quantitative results were normalized relative to the water control.

## Results

### Survival and hatching

**Fig. 2:**
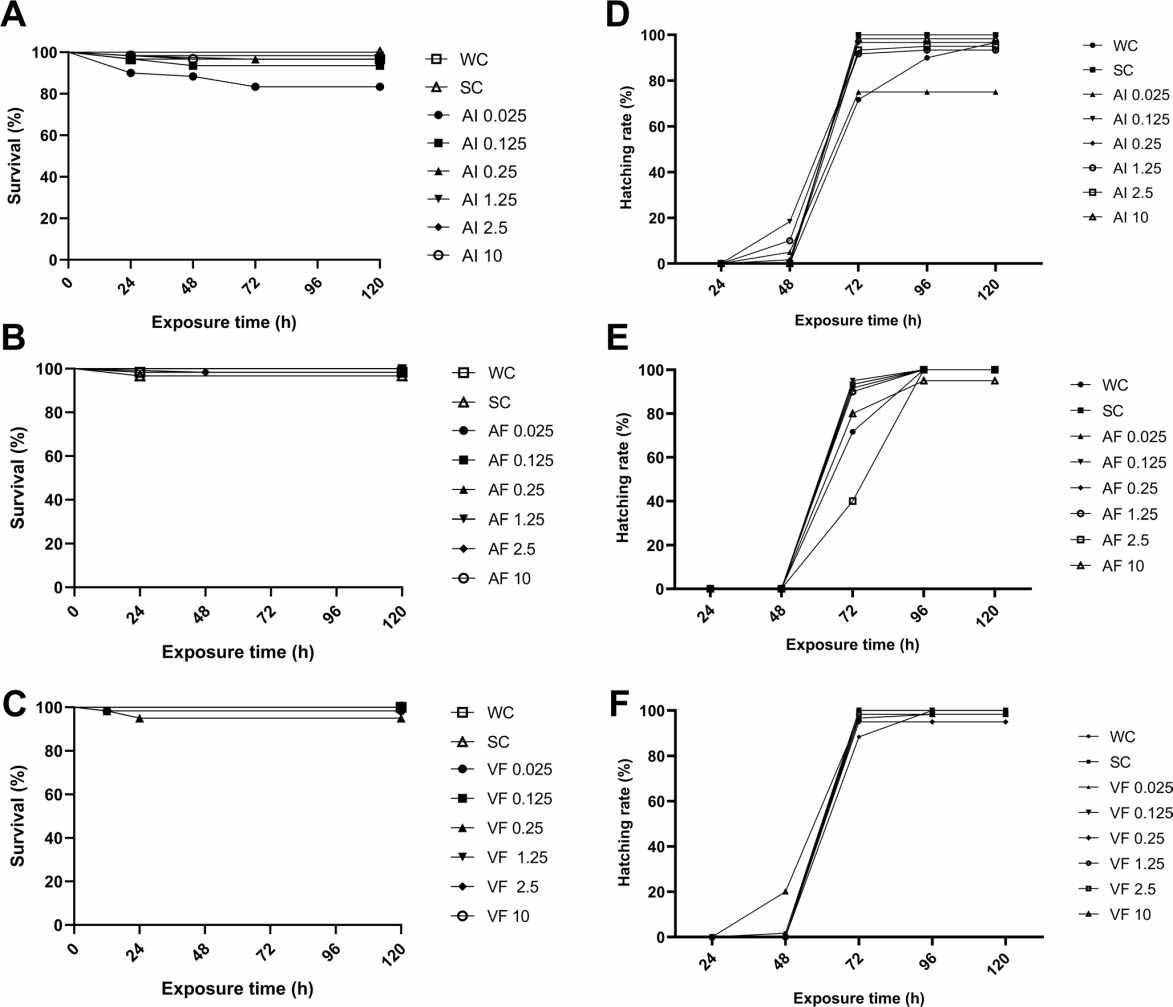
Survival rate (**A**-**C**) and hatching rate (**D**-**F**) of zebrafish embryos and larvae after 120 h of exposure to different concentrations of AI, AF, VF, and controls. WC = Water Control; SC = Solvent Control

None of the treatments affected embryo survival throughout the exposure period compared to the controls (*p* > 0.05) (Fig. 2A-C). The AI and the other PPFs did not impact larval hatching (Fig. 2D-F), since most of the larvae had hatched between 72 and 96 hpf, which is within the normal hatching period for zebrafish.

### Teratogenicity

**Fig. 3:**
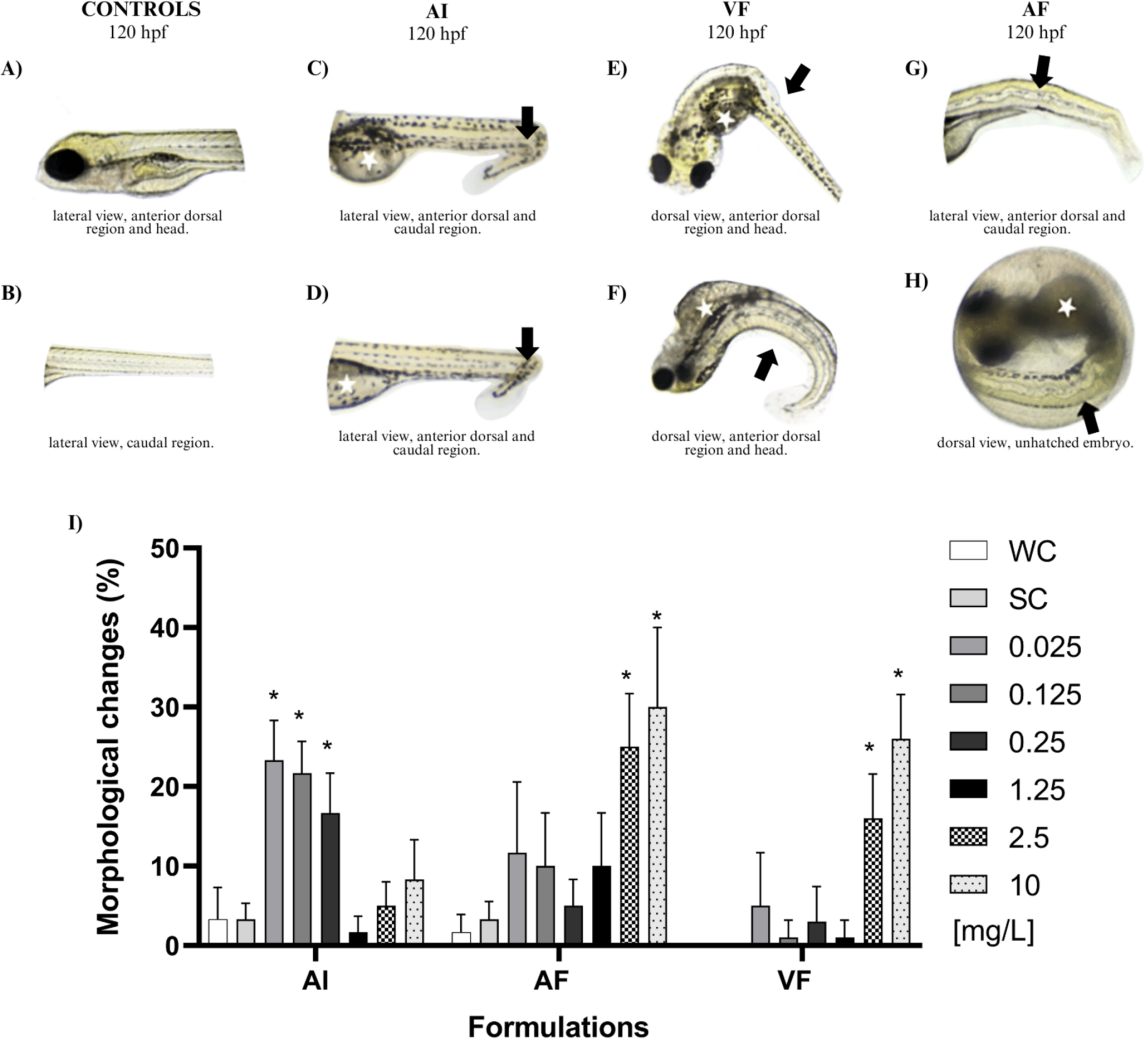
Main morphological alterations (**A**-**H**) and frequency of morphological changes (**I**) observed in zebrafish larvae after exposure for 120 h to different concentrations of AI, AF, VF, and controls. AF induced more pronounced morphological alterations compared with VF. Long arrow: Notochord malformation; White star: Opaque and poorly reabsorbed yolk sac. WC = Water Control; SC = Solvent Control. *Different from controls (*p* < 0.05)

Several morphological abnormalities were observed in response to the treatments. The main malformations observed included notochord deformities and opacity and darkening of the yolk sac (Fig. 3C-H). Lower and intermediate concentrations of IA (0.025, 0.125, and 0.25 mg L^− 1^) induced more abnormalities compared to higher concentrations (*p* < 0.05). Interestingly, exposure to AF and VF exhibited an opposite response pattern, causing malformations at higher concentrations (2.5 and 10 mg L^− 1^) (Fig. 3-I). These results demonstrate the potential influence of the PPF ingredients on AI toxicity. Notochord deformities were observed after exposure to both the AI and the PPFs, with greater severity following AF exposure. In the VF group, alterations in yolk sac shape and color and notochord deformities were more frequent. Although the notochord changes varied in presentation, including distal tail curvature, kyphosis, lordosis, scoliosis, and notochord fractures, they were grouped under the category of notochord deformities for analysis. In the AI group, the most common findings were reduced yolk sac resorption or yolk sac darkening and opacity, along with mild distal notochord alterations. Less frequent effects included mild pericardial edema, eye malformations, and developmental delay.

### Body pigmentation analyses

**Fig. 4:**
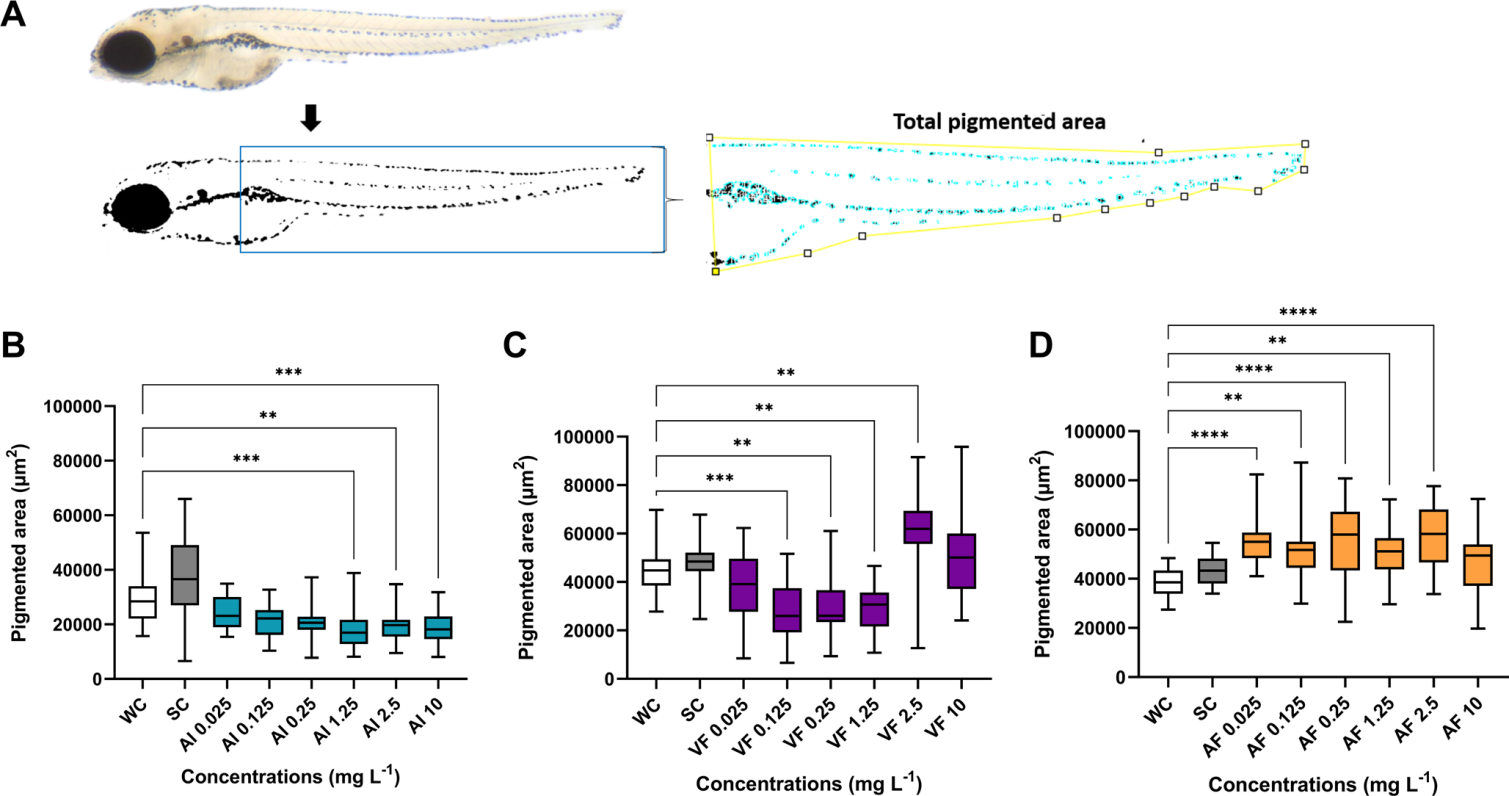
Body pigmentation analyses (**A**) measured in zebrafish larvae after exposure for 120 h to different concentrations of AI (**B**), VF (**C**), AF (**D**), and controls. AF and VF induced opposite effects on pigmentation at low and intermediate concentrations. WC = Water Control; SC = Solvent Control. *Different from controls (*p* < 0.05)

The total area occupied by melanocytes was quantified using ImageJ (Fig. 4-A). The results highlighted distinct effects of AI, AF, and VF on pigmentation. The AI caused a slight reduction in pigmentation at intermediate to higher concentrations (1.25–10 mg L^− 1^) (Fig. 4-B). Similarly, VF exhibited the most significant decrease at low to intermediate concentrations (0.125–1.25 mg L^− 1^) (Fig. 4-C). In contrast, AF significantly increased the pigmented area across most tested concentrations (0.025–2.5 mg L^− 1^) compared to the WC (Fig. 4-D). These results highlight the distinct effects between the AI and the commercial PPFs, with VF showing an opposite impact on zebrafish pigmentation compared to AI and AF.

### Morphometric analyses

**Fig. 5:**
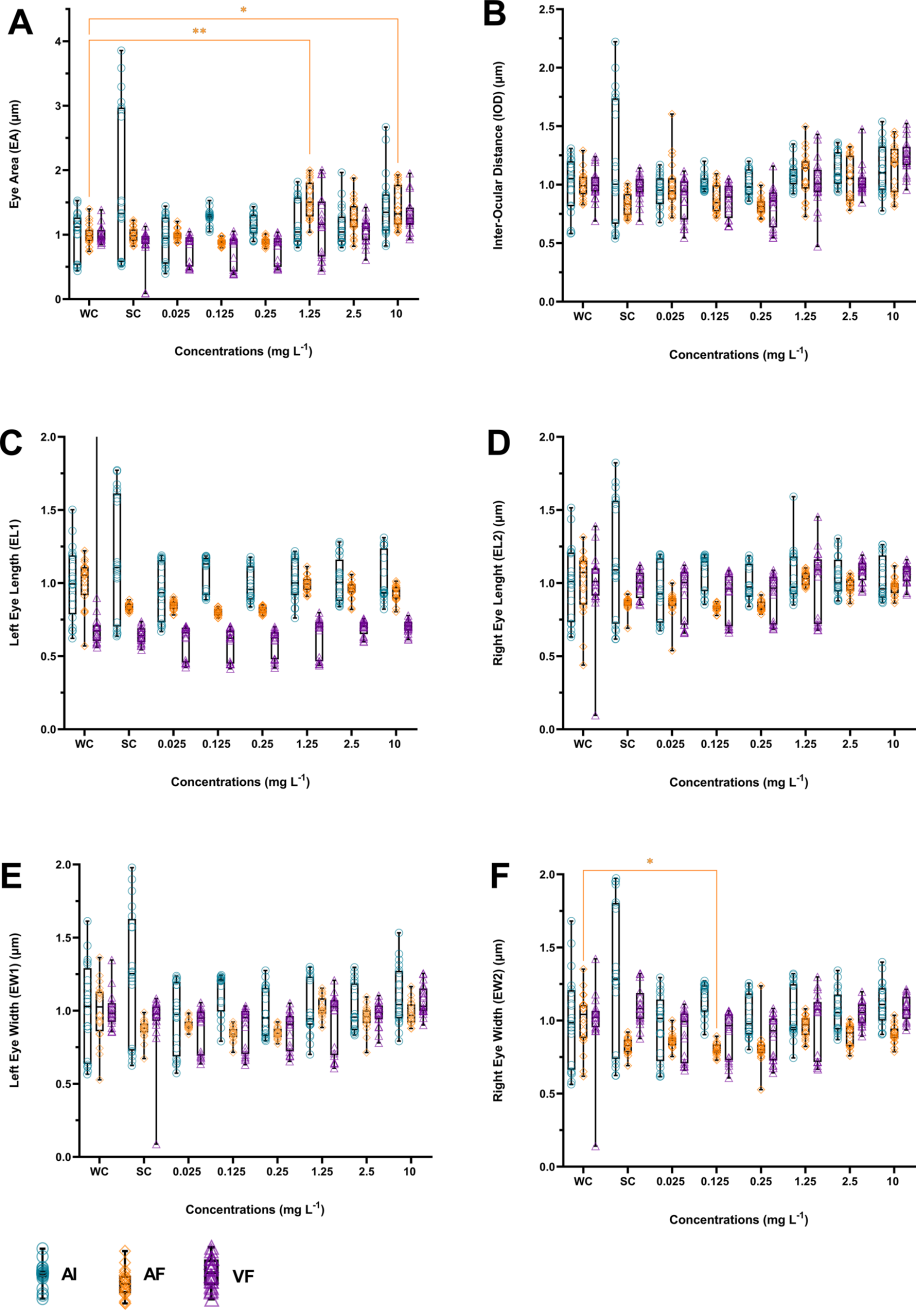

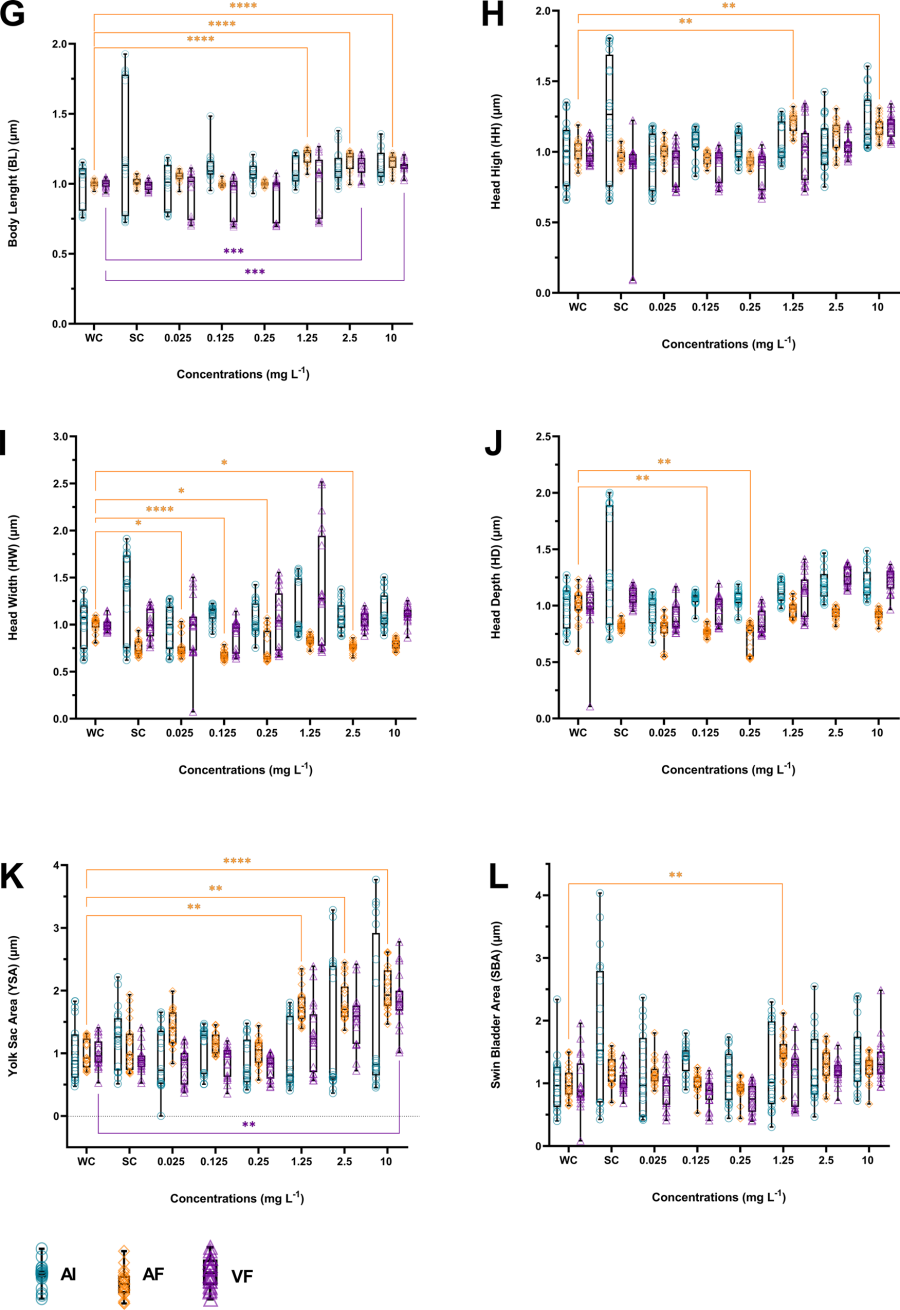
Morphometric analyses measured in zebrafish larvae after exposure for 120 h to different concentrations of AI, VF, AF, and controls. WC = Water Control; SC = Solvent Control. Eye area (**A**), Interocular distance (**B**), Left eye length (**C**), Right eye length (**D**), Left eye width (**E**), Right eye length (**F**), Body Length (**G**), Head high (**H**), Head width (**I**), Head depth (**J**), Yolk sac area (**K**), Swin bladder area (**L**). Blue represents the Active Ingredient (AI), purple represents the Veterinary Formulation (VF), and orange represents the Agricultural Formulation (AF). AF caused prominent increases in BL, YSA, HH, and reductions in HW and HD, whereas VF showed only mild increases in BL and YSA. *Different from WC (*p* < 0.05) **Different from WC (*p* < 0.01) ***Different from WC (*p* < 0.001) ****Different from WC (*p* < 0.0001)

Exposure of zebrafish larvae to the AF induced alterations primarily at intermediate (1.25 mg L^− 1^) and high (2.5 and 10 mg L^− 1^) concentrations, with some effects also observed at lower concentrations (0.025 and 0.125 mg L^− 1^). In contrast, VF exposure induced fewer and less pronounced alterations, with significant increases in BL at 2.5 and 10 mg L^− 1^ (Fig. 5-G), and in YSA at 10 mg L^− 1^ (Fig. 5-K). No other parameters were affected by VF at the tested concentrations. At the same time, no significant changes were observed following exposure to the AI (*p* < 0.05).

**Fig. 6:**
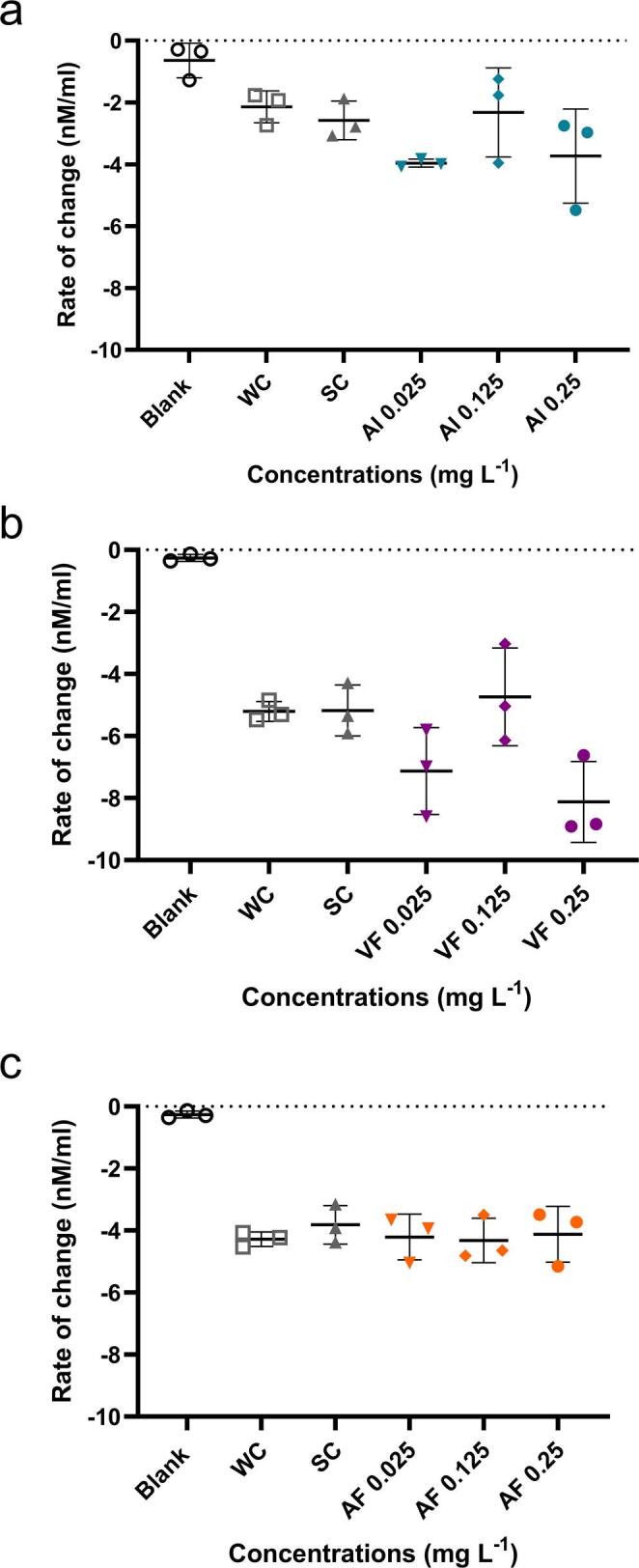
Oxygen consumption of zebrafish larvae after 168 h of exposure (**A**-**C**) to different concentrations of AI, AF, VF, and controls. WC = Water Control; SC = Solvent Control

In the sensory parameters, AF significantly increased EA at 1.25 and 10 mg L^− 1^ (Fig. 5-A) and reduced EW2 at 0.125 mg L^− 1^ (Fig. 5-F), with no significant effects on IOD, EL1, EL2, and EW1 (Fig. 5A-E). Regarding physiological parameters, YSA increased at 1.25, 2.5, and 10 mg L^− 1^ (Fig. 6-K), while SBA increased only at 1.25 mg L^− 1^ (Fig. 5-L). Skeletal changes included increased BL at 1.25, 2.5, and 10 mg L^− 1^ (Fig. 5-G). For muscle parameters, HH increased at 1.25 and 10 mg L^− 1^, whereas HW was significantly reduced at 0.025, 0.125, 0.25, and 2.5 mg L^− 1^; and HD at 0.125 and 0.25 mg L^− 1^ (Fig. 5H-J).

Most alterations for both PPFs were observed at intermediate and high concentrations (1.25 and 2.5 mg L^− 1^), suggesting a threshold of physiological sensitivity. Overall, AF elicited more extensive and pronounced morphometric effects than VF and AI (*p* < 0.05). These findings indicate differential effects between AI and its commercial PPFs, with AF exhibiting greater toxicity.

### Oxygen consumption

The results obtained from the oxygraph indicated that, while there appeared to be a trend toward increased basal oxygen consumption following exposure to AI and VF, no significant differences were observed compared to the control group (Fig. 6A-C).

## Discussion

The low toxicity of AI to non-target aquatic organisms is attributed to its limited water solubility (0.08 mg L^− 1^ at 20 °C, pH 7). Although diflubenzuron is considered practically non-toxic to avian species, small mammals, freshwater and marine/estuarine fish (USEPA 1997; Sun et al. 2015), studies have reported adverse effects on different developmental stages of zebrafish at environmentally relevant concentrations (ng L^− 1^ - µg L^− 1^). For instance, Teixeira et al. (2024) have reported that exposure to 0.025 and 0.125 mg L^− 1^ of the AI resulted in behavioral alterations (impaired avoidance behavior) and increased production of ROS in zebrafish embryos. Another recent study conducted with zebrafish embryos chronically exposed (30 d) to 0.025 and 0.125 mg L^− 1^ the AI demonstrated reduced locomotor parameters in the new tank and aggression tests, while it induced hyperactivity in the social behavior test (Teixeira et al. 2025). The authors also reported significant changes in the mRNA levels of different genes in the brain, presenting a reduction in *nfe2l2* gene expression and increased pro-inflammatory gene expression (*tnf-α* and *il-6*) at 0.125 mg L^− 1^, which reflected behavioral changes. Therefore, Fischer and Hall Jr (1992) emphasized that DFB, as a chitin synthesis inhibitor, induces both lethal and sublethal toxicity in non-target aquatic organisms, such as fish and invertebrates, even at environmentally relevant concentrations.

The limited availability of studies on the toxicity of DFB-based PPFs on embryotoxicity constrains the comprehensive risk assessment. In the literature, most works focus on different developmental stages. Draredja-Beldi and Soltani (2001) demonstrated that chronic exposure of *Gambusia affinis* juveniles (7 days old) to a DFB-based PPF at 0.000078 mg L^− 1^ for 45 days led to significant reductions in somatic growth, including body size and weight. In adult fish, Pereira-Maduenho and Martinez (2008) reported hematotoxic effects induced by DFB-based PPF (Dimilin^®^) at 25 mg L^− 1^ (of AI), characterized by decreased erythrocyte count and hemoglobin levels, activation of antioxidant defenses (GST and catalase), and inhibition of acetylcholinesterase (AChE) activity after 96 h of exposure. Abe et al. (2019) demonstrated that sublethal concentrations of a DFB-based PPF induced weight loss in *Hyphessobrycon eques* at levels 272-fold lower than the LC_50_ (10–12 mg L^− 1^), alongside histopathological damage, including gill edema, aneurysms, and hepatocellular hypertrophy.

In the present study, we demonstrated that AI and its commercial DFB-based PPFs, although not inducing significant lethality, elicited morphological abnormalities during the early developmental stages of zebrafish. Consistent with previous findings, exposure to the DFB AI at concentrations up to 10 mg L^− 1^ did not cause mortality in the embryos (Teixeira et al. 2024).

In this work, the increased toxicity observed in zebrafish larvae exposed to DFB-based PPFs (AF and VF), compared to the AI alone, may suggest a formulation-dependent modulation of bioavailability and biological effects. Given that AI has low water solubility and moderate lipophilicity (log Kow ≈ 3.8) (Fischer and Hall Jr 1992), the inclusion of surfactants and other co-formulants could potentially enhance its solubilization, environmental dispersion, and interaction with biological membranes. These changes might contribute to increased uptake in aquatic organisms and, consequently, more pronounced toxic effects.

PPFs represent cocktails that contain one or more active ingredients alongside other substances, known as inert, adjuvants, or co-formulants, which improve the dissolution, stability, absorption, and pesticidal action of the AI (Cox and Surgan 2006; Nagy et al. 2020). Therefore, depending on the formulation, the toxicity may change. For instance, according to Seaman (1990), emulsifiable concentrate formulations can be more dermallytoxic and irritant, while emulsions in water can reduce dermal irritation and toxicity over emulsifiable concentrate.

Furthermore, due to its hydrophobic nature, diflubenzuron tends to associate with organic matter (USEPA 2019) and particles (An et al. 2024). We observed the presence of water- and solvent-insoluble particles in VF and water-insoluble particles in AF, which could influence DFB’s availability and lead to slightly different responses in terms of morphological alterations and pigmentation when compared to the AI. However, in this study, we do not have information on the formulation’s composition, and therefore cannot determine how each component may affect AI’s availability and toxicity. According to Straw (2024), studies related to the impacts of co-formulations are limited by EU and US law, which protects the ingredient list as a secret from the public and researchers, which limits the capacity to understand the potential adverse effects on non-target organisms.

The role of adjuvants and additives in commercial formulations remains a critical factor for consideration. Nagy et al. (2020) performed a systematic review comparing the toxicity of pesticide AI and their PPFs. Most of the studies (24 of 36) reported increased toxicity of the PPFs when compared to their AI, which, in most cases, were attributed to the presence of adjuvants in the formulations. According to the authors, these findings emphasize the inadequacy of current testing requirements for PPFs. These requirements mainly focus on assessing individual ingredients and at least one representative use and formulation. They often overlook the potential risks arising from interactions between active ingredients and other components in various commercial pesticide formulations. This oversight may lead to misinterpretation of the toxicological profile of these products.

The melanin pathway is highly conserved among vertebrates. Melanocyte-stimulating hormones (MSHs) are the primary regulators of melanin type and production and of melanosome biogenesis, particularly through α-MSH (Nilsson Sköld et al. 2013). Melanocyte concentrating hormones (MCHs) also play an important role in controlling the dispersion and aggregation of melanosomes in teleost fish (Bertolesi et al. 2019; McNamara et al. 2021). The role of DFB in pigmentation alteration has been scarcely explored in the literature over the past decades. In the early 1980s, Norman and Meola (1983) described DFB’s effect on inhibiting melanogenesis in mouse melanoma cells (B16-F1) after three 72 h passages, as well as its impact on replication capacity. The authors attributed the inhibition of melanogenesis to alterations in the permeability of the cytoplasmic membrane. Although the melanin pathway and its regulatory mechanisms are well conserved among vertebrates (McNamara et al. 2021), it is difficult to extrapolate DFB’s mechanisms of action on melanogenesis in vitro to healthy cells of zebrafish embryos. Therefore, its effect on melanogenesis in zebrafish embryos remains poorly explored.

Evidence shows that a commercial formulation containing DFB can disrupt respiratory physiology in fish. The study by Pereira-Maduenho and Martinez (2008) demonstrated that exposure of *Prochilodus lineatus* to this formulation reduced erythrocyte counts and hemoglobin levels. The authors also reported hyperglycemia and elevated acetylcholinesterase activity, suggesting neurophysiological disturbance and increased energetic demand. Recent studies support these patterns across species. Pesticides such as the pyrethroid fenpropathrin increase oxygen consumption and impose metabolic strain in zebrafish (Saputra et al. 2023). Additional evidence shows that contaminants alter energy allocation and metabolic regulation even in early developmental stages (Diogo et al. 2025). Fish consistently adjust oxygen consumption under chemical or environmental stress, reinforcing the sensitivity of metabolic endpoints (Onukwufor and Wood 2020). In contrast, the present study detected no changes in larval oxygen consumption or yolk sac size after exposure to either the AI or its PPF’s. These results suggest that, under the tested conditions, DFB may not have elicited measurable respiratory or metabolic stress in zebrafish larvae. This outcome cautiously refines current understanding by indicating that early-life metabolic sensitivity to DFB could be lower than previously assumed and may vary according to species, developmental stage, exposure duration, and environmental context.

### Limitations and perspectives

Although our results revealed important differences between the toxicity of the PPFs and the AI, several limitations should be considered. The low solubility of DFB likely plays a significant role in the toxicity of both the AI and the PPFs. Because this study evaluated nominal concentrations, the actual exposure levels may have been lower than intended due to limited AI solubility. Future studies should therefore measure actual AI concentrations and examine how different formulations influence its solubility.

Additional research is needed to clarify the molecular mechanisms underlying DFB toxicity and to determine whether the observed morphological changes can be interpreted within established adverse outcome pathways. Toxicity may also be affected by exposure conditions such as duration, medium renewal, temperature, organism model, and developmental stage, and these factors should be carefully considered before extrapolating the results.

For regulatory purposes, complete formulations are not tested for long-term toxicity by industry applicants. Historically, neglecting the potential toxicity of co-formulants during pre-market testing has resulted in the entry into the market of pesticides containing co-formulants with problematic toxicity profiles (Robinson et al. 2020). Furthermore, the lack of information on unidentified components present in the formulations is an additional limitation in assessing their toxicities. Advances in chemical analyses to characterize the constituents of formulations, particularly in widely used pesticides, would represent a significant contribution to ecotoxicology.

## Conclusions

The present study demonstrates that while the AI and its commercial PPFs do not affect zebrafish embryo survival or hatching rates, they elicit significant sublethal effects, morphological abnormalities, pigmentation alterations, and morphometric parameters. Based on our findings, we hypothesize that the formulation modulates toxicity by altering the solubility of AI. In this study, the AF and VF caused more morphological abnormalities at the highest concentrations, while the AI showed similar effects at lower concentrations. Similarly, we demonstrated that the two DFB-based PPFs can induce distinct effects on larval body pigmentation, underscoring the formulation’s influence on toxicological outcomes. Our findings align with prior research identifying DFB’s AI role as a chitin synthesis inhibitor, which induces sublethal toxicity in non-target aquatic organisms. However, commercial DFB-based PPFs appear to change these effects, possibly due to the presence of adjuvants, other additives, and particulates. The observed differences in toxicity between AI and its PPFs underscore the necessity of evaluating not just the AI but also the full PPFs, as their interactions can lead to unanticipated toxicological outcomes. This study emphasizes the need for regulatory frameworks to account for the unique toxicological profiles of commercial PPFs. Future work should focus on identifying co-formulants and degradation products to clarify formulation-dependent toxicity.

## Data Availability

The datasets generated during and/or analyzed during the current study are available from the corresponding author on reasonable request.
